# Balancing accuracy and user satisfaction: the role of prompt engineering in AI-driven healthcare solutions

**DOI:** 10.3389/frai.2025.1517918

**Published:** 2025-02-13

**Authors:** Mini Han Wang, Xudong Jiang, Peijin Zeng, Xinyue Li, Kelvin Kam-Lung Chong, Guanghui Hou, Xiaoxiao Fang, Yang Yu, Xiangrong Yu, Junbin Fang, Yi Pan

**Affiliations:** ^1^Zhuhai People's Hospital (The Affiliated Hospital of Beijing Institute of Technology, Zhuhai Clinical Medical College of Jinan University), Zhuhai, China; ^2^Department of Ophthalmology and Visual Sciences, The Chinese University of Hong Kong, Kowloon, Hong Kong SAR, China; ^3^Faculty of Data Science, City University of Macau, Taipa, Macao SAR, China; ^4^Zhuhai Institute of Advanced Technology Chinese Academy of Sciences, Zhuhai, China; ^5^Digital Medicine and Artificial Intelligence Association, Macau, Macao SAR, China; ^6^Beijing Normal University - Hong Kong Baptist University United International College, Zhuhai, China; ^7^Perspective Technology Group, Zhuhai, China; ^8^School of Optometry & Ophthalmology, Tianjin Medical University, Tianjin, China; ^9^Zhuhai Aier Eye Hospital, Zhuhai, China; ^10^Department of Optoelectronic Engineering, Jinan University, Shenzhen, China; ^11^Key Laboratory of Intelligent Bioinformatics, Shenzhen Institute of Advanced Technology, Shenzhen, China

**Keywords:** internet of things (IoT), artificial intelligence (AI), dry eye disease (DED), prompt engineering, healthcare virtual assistant, generative pre-trained transformer-4 (GPT-4)

## Abstract

**Introduction:**

The rapid evolution of the Internet of Things (IoT) and Artificial Intelligence (AI) has opened new possibilities for public healthcare. Effective integration of these technologies is essential to ensure precise and efficient healthcare delivery. This study explores the application of IoT-enabled, AI-driven systems for detecting and managing Dry Eye Disease (DED), emphasizing the use of prompt engineering to enhance system performance.

**Methods:**

A specialized prompt mechanism was developed utilizing OpenAI GPT-4.0 and ERNIE Bot-4.0 APIs to assess the urgency of medical attention based on 5,747 simulated patient complaints. A Bidirectional Encoder Representations from Transformers (BERT) machine learning model was employed for text classification to differentiate urgent from non-urgent cases. User satisfaction was evaluated through composite scores derived from Service Experiences (SE) and Medical Quality (MQ) assessments.

**Results:**

The comparison between prompted and non-prompted queries revealed a significant accuracy increase from 80.1% to 99.6%. However, this improvement was accompanied by a notable rise in response time, resulting in a decrease in SE scores (95.5 to 84.7) but a substantial increase in MQ satisfaction (73.4 to 96.7). These findings indicate a trade-off between accuracy and user satisfaction.

**Discussion:**

The study highlights the critical role of prompt engineering in improving AI-based healthcare services. While enhanced accuracy is achievable, careful attention must be given to balancing response time and user satisfaction. Future research should optimize prompt structures, explore dynamic prompting approaches, and prioritize real-time evaluations to address the identified challenges and maximize the potential of IoT-integrated AI systems in medical applications.

## Highlights

- IoT-integrated AI enhances Dry Eye diagnosis accuracy by over 19%, though response time increases.
- Proposed prompt engineering significantly boosts AI precision in healthcare, improving decision-making quality.
- The study lays the groundwork for future AI advancements in ophthalmology, prioritizing accuracy and patient outcomes.

## 1 Introduction

The emergence of expansive language models (LLMs) (Kasneci et al., 2023), exemplified by the generative pre-trained transformer-4 (GPT-4) (Biswas, 2023), signifies a pivotal era in artificial intelligence (AI). While the diverse applications of GPT-4 have been extensively investigated, its latent potential in the medical domain, particularly within the realm of ophthalmology, represents an unexplored frontier. This study aims to fill this gap by examining GPT-4’s effectiveness in recognizing rare ophthalmic diseases across a spectrum of simulated scenarios involving diverse end-users, encompassing patients, family physicians, and junior ophthalmologists.

Within the expansive landscape of AI applications, the rise of virtual assistants (Rehman et al., 2020), particularly those anchored in robust language models like ChatGPT, has garnered significant attention. ChatGPT, an AI-powered chatbot, has demonstrated its efficacy across various domains, notably in medicine, showcasing competence in generating thorough lists of differential diagnoses, structuring patient notes, and providing accurate responses to queries related to retinal diseases. Nevertheless, the effective deployment of these virtual assistants relies crucially on the enhancement of input queries, referred to as “prompts,” achieved through the strategic implementation of prompt engineering (Wu and Hu, 2023).

Prompt engineering, as a nuanced procedure, entails the meticulous formulation and refinement of prompts to direct LLMs in producing outputs that align with user expectations. The importance of prompt engineering resides in its capacity to enhance the efficacy of LLMs, explore innovative applications, and optimize resource utilization. Through skillful prompt construction, both information seekers and developers can fully harness the capabilities of LLMs, customizing solutions to address prevalent challenges in output generation and interaction (Azimi et al., 2025).

The overarching objective of this research is to propel the user experience within ophthalmic virtual assistants through the cultivation of innovative prompt design. A comprehensive understanding of prompt engineering not only bolsters the development of sophisticated and efficient LLM applications but also contributes substantively to the advancement of natural language processing (NLP) tasks (Nath et al., 2022), ultimately enriching the functionalities of conversational AI systems. As prompt engineering ascends in prominence, it stands poised to assume an increasingly pivotal role in unlocking the full potential of LLMs in the foreseeable future. This trajectory is anticipated to spawn new employment opportunities for adept prompt engineers, concurrently enabling information seekers and developers to experiment with advanced prompts, culminating in the creation of user interfaces that afford enhanced control over LLM outputs, thereby unlocking hitherto inconceivable applications.

Dry Eye Disease (DED) (Wang et al., 2024b), a prevalent ocular condition (Wang et al., 2024a; Zhou et al., 2024), serves as the focal point in this study, which endeavors to simulate 5,747 patient complaints. Employing the APIs of OpenAI GPT-4.0 and ERNIE Bot-4.0, the research establishes a DED-detection-prompt system designed to assess the urgency of seeking medical attention. This aims to elevate the accuracy of LLM responses, enhancing the interpretability of interactions between DED and AI machines. Additionally, a Bidirectional Encoder Representations from Transformers (BERT) machine learning model, based on the responses generated by LLMs, is employed for text classification (urgent and non-urgent). This model plays a crucial role in evaluating the quality of DED-related question answering. Furthermore, the LLM-generated inquiries are assessed through the lens of user satisfaction, considering both the satisfaction of experiences and medical quality. The study underscores the pivotal role of prompt engineering in shaping user interactions with virtual assistants within the developmental landscape of ophthalmic GPT. By elucidating the mechanics of prompt engineering and its contribution to the creation of precise, context-specific, and nuanced responses from LLMs, this research aims to empower information seekers and developers to effectively leverage the capabilities of GPT-4.0.

## 2 Literature review

Given the challenge posed by a scarcity of biomedical data for domain-specific pretraining, alternative methodologies involve fine-tuning with meticulously curated medical and scientific text (Wang et al., 2022). Prompting strategies, including chain-of-thought (CoT) prompting (Wei et al., 2022) and retrieval augmentation, exhibit promise in enhancing domain-specific performance without requiring substantial computational and economic investments. These strategies, resembling domain-specific fine-tuning mechanisms on a broader scale, have the potential to advance ophthalmic virtual assistants (Iskander et al., 2021) by mitigating inherent limitations and optimizing their utility in clinical contexts. As highlighted by Tan et al. (2023), human expert annotation of CoT explanations generated by LLMs in medical question-answering tasks yields promising results, showcasing sound reasoning, thought processes, knowledge recall, and comprehension of both the question and context. Further avenues for improvement include exploring features such as uncertainty-aware applications that provide probability scores for generated responses, offering alternative recommendations for low-probability scenarios, and reporting the differential weights of input tokens contributing to the generated answer. These endeavors contribute to enhancing the transparency and reliability of ophthalmic virtual assistants in the context of GPT-based development (Tan et al., 2023).

Zandi et al. (2024) conducted a comprehensive evaluation and comparative analysis of GPT-4 and Bard, specifically examining their responses to prevalent ophthalmic complaints. The assessment encompassed simulated patient vignettes, including 40 critical diagnoses, and targeted inquiries designed to elucidate proposed diagnoses and triage recommendations. The study also explored the influence of prompt descriptiveness on response quality, aiming to enhance our comprehension of how these advanced language models can be optimally employed in future patient-oriented contexts. Systematically constructing common scenarios encountered by patients in ophthalmology, the research distributed identified diagnoses across distinct ophthalmic specialties, further enriching the study’s complexity. Urgency levels for seeking care were assigned to each scenario. The methodical approach involved creating two prompts for each diagnosis, varying in the number of key clinical descriptors. Collaboration with experienced ophthalmologists ensured consensus on the intended diagnosis and urgency level for each patient scenario. Employing GPT-4 and Bard, the experiments were conducted with standardized prompts entered into AI chatbots, strategically designed to address differential diagnoses, leading diagnoses, and provide triage recommendations. Anticipated to offer valuable insights, the study’s findings have the potential to inform the implementation of conversational AI in delivering timely, accurate, and secure ophthalmic health information, thereby meeting the needs of patients and facilitating informed decision-making (Zandi et al., 2024).

In summary, the current scholarly discourse has scrutinized the evaluation of LLMs within the context of ophthalmology, acknowledging their growing relevance. While foundational LLMs have exhibited the ability to generalize across diverse domains in general NLP tasks, their performance in ophthalmology has been inconsistent, yet suggestive of potential applications in eye healthcare. To address this, strategies have been proposed to enhance foundational LLMs for clinical tasks, including constructing domain-specific LLMs through pretraining with carefully curated medical text, fine-tuning using domain-specific medical data, and employing innovative prompting strategies. Furthermore, the development of ophthalmic virtual assistants utilizing GPT-based models raises concerns about the inherent “black-box” nature of LLMs, obscuring the decision-making process and compromising interpretability (Wang et al., 2023a). This lack of explainability (Wang et al., 2023b,c) becomes particularly problematic in healthcare (Wang et al., 2023a), where the credibility of generated responses is crucial, and misinformation can have severe consequences. Notably, responses from LLMs often lack supporting citations or information sources, further challenging their reliability. Therefore, a more diverse array of prompts offers a significant advantage to accommodate potential variations in user input. By structuring prompts to solicit step-by-step reasoning, analogous to an expert ophthalmologist outlining a differential diagnosis, virtual assistants can offer insights into their decision-making process alongside the final answer.

## 3 Materials and methods

The documentation for ethical clearance in this research was obtained from the ethics review board at Zhuhai People’s Hospital. Data, acquired in both English and Chinese languages, was gathered using the application programming interfaces (APIs) of GPT-4.0 and ERNIE Bot-4.0, which are accessible to the public through a paid subscription. In contrast, Bard was freely accessible for use. This study has chosen DED (Wang et al., 2024a), a prevalent ocular condition, as a case study to investigate the progress of ophthalmic virtual assistants. The primary focus is on expeditious engineering strategies designed to enhance user interaction within the framework of GPT-based development. Prompt design played a critical role in enhancing the performance and reliability of the AI-driven system for diagnosing DED. The following techniques were employed in the prompt design process to ensure that the AI models (GPT-4.0 and ERNIE Bot-4.0) generated accurate, context-specific, and clinically relevant responses.

### 3.1 Simulated patient prompt conduction in DED

The prompt design process was highly iterative, involving continuous testing and refinement to improve the effectiveness of the prompts. Initially, a set of baseline prompts was developed based on domain-specific knowledge and the requirements of the DED diagnosis process. These prompts were then tested with the AI models to observe their performance. Feedback from the AI’s responses was analyzed to identify areas for improvement. This feedback loop allowed us to refine the prompts iteratively, adjusting the wording, structure, and specificity to align better with the desired outcomes. For example, prompts that led to ambiguous or irrelevant responses were rephrased to be more direct and focused, improving the overall accuracy of the AI’s outputs.

To ensure that the prompts were effective across diverse demographics and clinical scenarios, we emphasized contextual relevance in the prompt design. Each prompt was carefully crafted to include key medical terms, patient symptoms, and diagnostic criteria relevant to DED. This approach ensured that the AI could interpret and respond to the prompts accurately within the specific context of ophthalmic healthcare. We also customized prompts for different patient profiles and clinical scenarios, recognizing that patient demographics (e.g., age, gender, lifestyle) and the variability of symptoms could influence the diagnosis. Multiple versions of each prompt were created to cater to these differences, ensuring that the AI could provide appropriate responses regardless of the patient’s background or the complexity of the case.

This study employed advanced prompt engineering strategies, such as Chain-of-Thought prompting, to enhance the AI’s reasoning capabilities. Chain-of-Thought prompting involves designing prompts that encourage the AI to think through a problem step by step, leading to more thorough and accurate responses. This strategy was particularly effective in complex diagnostic scenarios where multiple factors needed to be considered. Additionally, we used role-playing prompts to instruct the AI to take on the role of a dry eye specialist. This approach helped the AI generate responses that were more aligned with expert clinical advice, improving the reliability and relevance of the information provided to users.

The prompts were designed using a structured approach, where each prompt guided the AI through a logical sequence of diagnostic questions. This step-by-step structure was crucial for maintaining the coherence and relevance of the AI’s responses. The structure typically began with a general inquiry about the patient’s symptoms, followed by more specific questions aimed at identifying potential causes, necessary treatments, and urgency levels. For example, a prompt sequence might start with, “Describe any symptoms of eye discomfort you are experiencing,” followed by, “Based on these symptoms, what could be the underlying causes of Dry Eye Disease?” This structured design ensured that the AI’s responses were not only accurate but also aligned with the clinical workflow.

**Figure 1:**
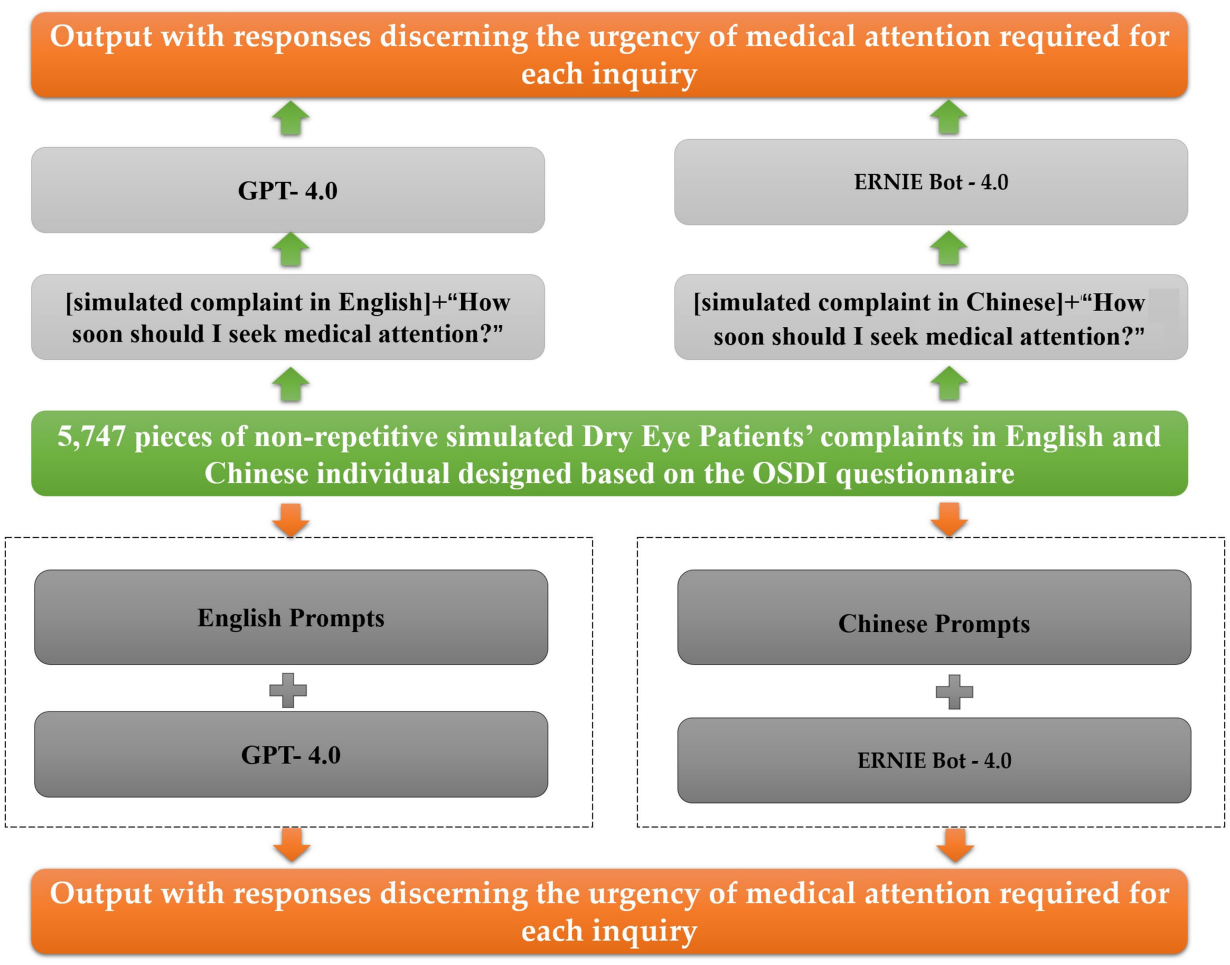
Scheme of overall study design of LLM prompts of AI DED detection.

This research systematically developed patient complaints related to DED using descriptors derived from the Ocular Surface Disease Index (OSDI) questionnaire (Zhang et al., 2021), as outlined in Supplementary Tables S1, S2. The OSDI, developed by Allergan Inc., Irvine, CA, USA, is a widely employed instrument for evaluating DED, consisting of 12 items with a final score ranging from 0 (no symptoms) to 100 (severe symptoms) points. The prompt for the diagnosis of DED in the clinic was established by comparing it to a GPT query without a prompt (Figure 1). A descriptor, in this context, is defined as a clinically relevant piece of information addressing aspects such as relevant history, onset, duration, laterality, specific ocular anatomy, vision, dyschromatopsia, pain, photophobia, visual disturbances, or any other clinical characteristic. Urgency levels for seeking care were determined based on discussions with experienced ophthalmologists (P.B., A.E.B., and R.C.B), with urgency categorized as same day, urgent (<1 week) (for descriptions categorized as occasionally/Never), or non-urgent (>1 week) (for descriptions categorized as One or more descriptions is often like this/most of the time/often/half of the time). Supplementary Tables S3, S4 provide predicted answer results (with and without prompt) for simulated complaints of Dry Eye Patients in English and Chinese, respectively.

### 3.2 AI-driven inquiry by utilizing GPT-4.0 and ERNIE Bot-4.0 LLM APIs

The selection of GPT-4.0 for handling English queries and ERNIE Bot-4.0 for addressing Chinese queries is grounded in the distinct strengths of these models in their respective linguistic domains. GPT-4.0, developed by OpenAI, has been extensively trained on a vast corpus of English-language data, rendering it highly proficient in understanding, generating, and responding to English text. In contrast, ERNIE Bot-4.0, developed by Baidu, is specifically optimized for Chinese language processing, drawing upon its deep understanding of Chinese grammar, syntax, and cultural nuances. This design strategy ensures that each model functions within its optimal linguistic environment, thereby maximizing the accuracy and relevance of the responses generated for users in both languages. By deploying GPT-4.0 for English and ERNIE Bot-4.0 for Chinese, we leverage the specialized capabilities of each model to effectively manage the complexities of their respective languages, which enhances the overall performance of the system.

Given that GPT-4.0 and ERNIE Bot-4.0 are tailored for different languages—English and Chinese, respectively—direct comparison of their performance is both challenging and not entirely meaningful. The criteria typically used for comparison, such as accuracy, response time, and user satisfaction, are inherently influenced by the specific language each model processes. Since each model is optimized for excellence within its own linguistic domain, their performances are designed to be effective within those domains and are not directly comparable across languages. Moreover, differences in linguistic structure, cultural context, and user expectations between English and Chinese may lead to variations in model performance, even when both models are highly effective within their specific contexts. Thus, while each model is assessed based on its ability to process and generate language within its respective domain, these evaluations are not directly comparable due to the intrinsic differences in the languages they are designed to handle.

APIs from GPT-4.0 and ERNIE Bot-4.0 were employed to input simulated patient complaints and prompts related to DED in both English and Chinese languages into AI chatbots. This process took place between February 2nd, 2024, and February 14th, 2024, to generate responses using LLMs. GPT-4.0 demonstrated notable proficiency in English text analysis, while ERNIE Bot-4.0 excelled in Chinese NLP tasks. Each prompt followed a standardized 9-part stepwise approach (Table 1), specifically designed for DED detection prompts. An example prompt used was, “I hope you can play the role of a dry eye doctor, search for triggers, and propose reasonable treatment methods for DED, including conventional therapy, safe medication, daily care, herbal therapy, and other natural therapies. When providing advice, please consider the patient’s age, lifestyle, and medical history. Please consider the patient’s age, lifestyle, and medical history when providing advice. Please consider the patient’s age, lifestyle, and medical history when providing advice.” This prompt was followed by queries such as “[simulated complaint] + ‘what are the possible causes?’” and “[simulated complaint] + ‘Is it possible to get worse and how?’” A meticulous approach was taken with a chatbot history reset before each 9-part entry, ensuring sequential questions aimed at addressing a differential diagnosis, establishing a leading diagnosis, and providing triage recommendations.

**Table 1:** The designed DED-detection-prompt.

Steps	English
1	“I request your expertise as a dry eye specialist to identify triggers and recommend appropriate treatment strategies for Dry Eye Disease (DED). This encompasses conventional therapy, safe medications, daily care practices, herbal therapy, and other natural remedies. When offering guidance, please consider the patient’s age, lifestyle, and medical history, ensuring a comprehensive understanding of these factors.”
2	[simulated complaint] + “what are the possible causes?”
3	“What of these is most likely?”
4	[simulated complaint] + “What type of eye condition do you believe I may be experiencing?”
5	“What is the most probable diagnosis for this condition?”
6	[simulated complaint] + “Is it possible to get worse and how?”
7	“Is it possible to get better itself?”
8	“Under what condition that I must see an eye doctor? “
9	[simulated complaint] + “How soon should I seek medical attention? “

### 3.3 Measurements of the LLM chatbot responses

To assess responses, this study employed the accuracy of predicting the urgency for patients to seek medical attention. Utilizing a machine learning approach with BERT, the study categorized generated data into two clusters, namely “urgent” and “non-urgent.” The pre-trained BERT model was sourced from PyTorch Hub, and the fine-tuning process involved the use of labeled data. One-fifth of the data was labeled by three independent researchers, and the final label was determined through a voting mechanism. The training dataset and validation dataset were established in a ratio of 4:1.


Pseudocode of text classification based on BERT model
# Import necessary libraries
import torch
from transformers import BertTokenizer, BertForSequenceClassification
from torch.utils.data import DataLoader, TensorDataset
from sklearn.model_selection import train_test_split
from sklearn.metrics import accuracy_score, roc_auc_score
from tqdm import tqdm
# Load pretrained BERT model and tokenizer
model_name = "bert-model";  specify the model name
tokenizer = BertTokenizer.from_pretrained(model_name)
model = BertForSequenceClassification.from_pretrained(model_name, num_labels=2)
# we have a labeled dataset with 'text' and 'label' columns
loading dataset from a local file
dataset = load_dataset()
# Split the dataset into training and testing sets
train_data, test_data = train_test_split(dataset, test_size=0.2, random_state=42)
# Tokenize and preprocess the text data
def preprocess_data(data, tokenizer):
    inputs = tokenizer(data['text'].tolist(), padding=True, truncation=True, return_tensors='pt', max_length=512)
    labels = torch.tensor(data['label'].tolist())
    return inputs, labels
train_inputs, train_labels = preprocess_data(train_data, tokenizer)
test_inputs, test_labels = preprocess_data(test_data, tokenizer)
# Create DataLoader for training and testing
train_dataset = TensorDataset(train_inputs['input_ids'], train_inputs['attention_mask'], train_labels)
test_dataset = TensorDataset(test_inputs['input_ids'], test_inputs['attention_mask'], test_labels)
train_dataloader = DataLoader(train_dataset, batch_size=8, shuffle=True)
test_dataloader = DataLoader(test_dataset, batch_size=8, shuffle=False)
# Set up training parameters
optimizer = torch.optim.AdamW(model.parameters(), lr=2e-5)
criterion = torch.nn.CrossEntropyLoss()
# Training loop
num_epochs = 3
device = torch.device("cuda")
model.to(device)
for epoch in range(num_epochs):
    model.train()
    total_loss = 0
    for batch in tqdm(train_dataloader, desc=f"Epoch {epoch + 1}/{num_epochs}"):
       input_ids, attention_mask, labels = batch
       input_ids, attention_mask, labels = input_ids.to(device), attention_mask.to(device), labels.to(device)
       optimizer.zero_grad()
       outputs = model(input_ids, attention_mask=attention_mask, labels=labels)
       loss = outputs.loss
       total_loss += loss.item()
       loss.backward()
       optimizer.step()
    average_loss = total_loss / len(train_dataloader)
    print(f"Epoch {epoch + 1}/{num_epochs}, Average Training Loss: {average_loss}")
# Evaluation on the test set
model.eval()
predictions = []
true_labels = []
with torch.no_grad():
    for batch in tqdm(test_dataloader, desc="Evaluating"):
        input_ids, attention_mask, labels = batch
        input_ids, attention_mask, labels = input_ids.to(device), attention_mask.to(device), labels.to(device)
       outputs = model(input_ids, attention_mask=attention_mask)
       logits = outputs.logits
       predictions.extend(torch.argmax(logits, dim=1).cpu().numpy())
        true_labels.extend(labels.cpu().numpy())
# Calculate evaluation metrics
accuracy = accuracy_score(true_labels, predictions)
roc_auc = roc_auc_score(true_labels, predictions)
print(f"Accuracy: {accuracy * 100:.2f}%")
print(f"AUC: {roc_auc * 100:.2f}%")


Moreover, the evaluation of patient-chatbot interactions includes the assessment of two indicators (Chen et al., 2020): the patient’s satisfaction with service experiences (SE) and the patient’s satisfaction with the medical quality (MQ) of the LLM. SE is gauged by posing the question, “Please assign a numerical score from 1 to 100 to rate your satisfaction with this experience. Do you believe the responses from the LLM were prompt, comprehensible, and engaging?” MQ is assessed by inquiring, “Please assign a numerical score from 1 to 100 to rate your satisfaction with this experience. Do you think the responses from the LLM were professional, beneficial, and addressed your concerns?” Interaction satisfaction is determined by computing the average of SE and MQ parameters. In this study, five individuals were recruited to engage with the prompts, interact with LLMs, and self-assess satisfaction levels based on SE and MQ indicators. The final score of satisfaction level is derived from the average value of these assessments.

### 3.4 Testing and validation

The effectiveness of the designed prompts was rigorously tested through simulations and real-world scenarios, using datasets that were randomly selected from a diverse population across various demographics and clinical scenarios. Specific datasets included a wide range of patient profiles, ensuring that the prompts were applicable to different age groups, genders, and lifestyle factors. The validation process involved testing the AI’s performance using these prompts across a variety of patient cases, focusing on key metrics such as accuracy, response time, and user satisfaction.

By evaluating the AI’s performance across this diverse set of scenarios, we were able to fine-tune the prompts to meet the required standards for clinical use. This iterative process ensured that the prompts were effective in guiding the AI to produce accurate and contextually appropriate responses, regardless of the variability in patient symptoms or demographic factors. The rigorous testing and validation across diverse patient profiles were critical in confirming the robustness and reliability of the prompts in real-world clinical applications.

## 4 Results and discussion

### 4.1 Results

A total of 5,747 non-repetitive simulated patient complaints in English and an equivalent number in Chinese were generated, designed based on the Ocular Surface Disease Index (OSDI) questionnaires. Of these, 5,624 English responses and 5,745 Chinese responses were deemed effective, while others resulted in an error response from the API due to rate limit issues ({"error_code":336501,"error_msg":"Rate limit reached for RPM","id":"as-pgzr4wdk3t"}).

For classification into “urgent” and “non-urgent,” 5,624 English responses and 5,745 Chinese responses were input into the pre-trained DETR model for fine-tuning. The training model achieved an accuracy of 98%, with an AUC of 96%.

The findings presented in Table 2 highlight significant enhancements in the prediction of urgency levels when prompted queries are contrasted with non-prompted counterparts, showcasing an increase in accuracy from 74.1 to 94.6%. However, this advancement is coupled with a notable escalation in response time, shifting from 0.84 s for non-prompted queries to 7.81 s for prompted queries. Noteworthy is the marginal decrease in the overall satisfaction level from 80.3 to 77.85, indicating a potential trade-off between accuracy and response time. A detailed breakdown of satisfaction, considering SE and MQ, exposes a conspicuous reduction in SE (from 95.5 to 64.0) and a substantial augmentation in MQ satisfaction (from 65.1 to 91.7) associated with prompted queries. These outcomes underscore the critical need to meticulously weigh the trade-offs inherent in accuracy, response time, and user satisfaction during the development and deployment of conversational AI systems in medical applications. A case with each prompted query’s generated inquiry by LLM for every prompt is elucidated in Supplementary Tables S5.

**Table 2:** Measurement indicators for inquiry generated by LLM with non-prompt and prompt processes.

**Indicators**	**Non-prompt**	**Prompt**
Accuracy of predicting the urgency level	74.1%	94.6%
Time-consuming/per request	0.84 s	7.81 s
Satisfaction level	80.3	77.85
SE	95.5	64.0
MQ	65.1	91.7

SE, Satisfaction Experiences; MQ, medical quality. The red marked parameter is regarded as the subject with a better performance.

### 4.2 Discussion

The integration of real-time data, adaptive learning, and multi-model inputs significantly enhances the performance and reliability of AI-driven diagnostic systems, particularly in the context of DED detection. Real-time data integration allows the system to continuously process current patient information and environmental factors, enabling timely adjustments to diagnostic recommendations and improving early detection. Adaptive learning mechanisms further refine the AI’s decision-making processes by incorporating new data and feedback from clinical interactions, thereby enhancing its ability to personalize responses and adapt to evolving patient demographics and emerging trends. Additionally, the use of multi-model inputs, which draw from various data sources such as patient-reported symptoms, environmental sensors, ocular images, and historical records, enriches the diagnostic process by providing a comprehensive, multi-faceted analysis. This holistic approach not only increases diagnostic accuracy but also supports proactive disease management. The combined use of these elements contributes to a more responsive and personalized healthcare system, with significant implications for improving patient outcomes and advancing clinical practice. Future research should continue to explore the potential of these technologies, focusing on enhancing data interoperability and developing sophisticated algorithms that further improve the synthesis and application of multi-model inputs in AI-driven healthcare.

This study leverages simulated patient complaints as a foundational approach for initial system evaluation. However, simulated datasets inherently lack the full spectrum of variability, complexity, and unpredictability present in real-world clinical environments. To address this limitation, future research should prioritize the implementation of real-world clinical trials and longitudinal user studies conducted across diverse healthcare contexts. Such evaluations would facilitate comprehensive testing under dynamic and multifactorial conditions, yielding critical insights into the system’s diagnostic accuracy, scalability, and user acceptance. Furthermore, real-world validation would enable the identification of context-specific challenges, guiding targeted system enhancements and strengthening its overall reliability and clinical applicability.

Furthermore, the current study primarily supports English and Chinese languages, expanding its multilingual capabilities remains a critical objective for future development. Achieving this requires employing language-specific fine-tuning to adapt the model to the unique linguistic nuances and cultural contexts of various target languages. Additionally, cross-lingual transfer learning presents a promising solution by leveraging knowledge from high-resource languages to enhance performance in under-resourced languages. This approach would enable the system to serve a broader global user base while maintaining high levels of accuracy and contextual relevance. Future research should prioritize the creation of comprehensive multilingual datasets and the integration of advanced natural language processing techniques, including multilingual embeddings and cross-lingual pretraining. These strategies would enhance the system’s scalability and adaptability, ensuring its applicability across diverse healthcare environments and promoting equitable access to AI-driven healthcare solutions worldwide.

Findings show that the proposed prompted queries demonstrated a substantial increase in accuracy, reaching 94.6% compared to 74.1% for non-prompted queries. This signifies a considerable strength in utilizing well-structured prompts for assessing urgency in DED cases. The prompted queries exhibited a notable improvement in MQ satisfaction (from 65.1 to 91.7), indicating a strength in providing more professional, useful, and issue-resolving responses to users. Moreover, broadening the system’s applicability beyond DED constitutes a pivotal avenue for future investigation. The proposed framework exhibits considerable potential for adaptation to additional ophthalmic conditions, such as glaucoma and cataracts, where early detection and timely medical intervention remain imperative. Task-specific model fine-tuning may facilitate the recognition of diverse ophthalmic indicators by employing transfer learning strategies to preserve previously acquired knowledge while assimilating new diagnostic features. Moreover, extending the system’s functionality to encompass broader healthcare applications would necessitate integrating multimodal data sources, including medical imaging, patient histories, and biometric records, enabling a more comprehensive diagnostic approach. The development of modular components targeting distinct medical conditions could enhance the system’s scalability and interoperability within integrated healthcare ecosystems. Future research should emphasize constructing adaptable models capable of seamless expansion, thereby ensuring sustained improvements in diagnostic precision and informed clinical decision-making.

However, a drawback observed was the increase in response time for prompted queries, from 0.84 s to 7.81 s. This extended response time could negatively affect user experience, particularly in scenarios where promptness is critical, such as in urgent medical inquiries. The study also identified a decrease in service experience satisfaction for prompted queries, dropping from 95.5 to 64.0, which suggests potential user dissatisfaction with the speed, clarity, and engagement of the responses. To address the challenge of increased response time, several mitigation strategies warrant consideration. First, optimizing prompt structures through the simplification of query formulations and the reduction of complexity could enhance system efficiency. Second, employing parallel processing techniques would facilitate the concurrent management of multiple patient queries, thereby decreasing overall wait times. Third, implementing caching mechanisms for frequently requested responses could accelerate handling of common inquiries, particularly for recurrent medical conditions or standardized diagnostic procedures. Furthermore, future research should explore the development of a hybrid model capable of dynamically balancing accuracy and response time. Such a system could adaptively alternate between prompted and non-prompted query modes depending on contextual factors such as query urgency and system load. Additionally, incorporating context-aware dialogue mechanisms would allow the system to maintain a more fluid and intuitive conversational flow by dynamically adjusting responses to match user expectations. Such improvements could help preserve high MQ scores while enhancing overall user satisfaction. Future research should explore the integration of personalization techniques and machine learning-driven adaptive modules to create a responsive, user-centered AI healthcare system. By integrating these strategies, the proposed system could better align with real-world clinical requirements while maintaining high diagnostic accuracy and user satisfaction.

Additionally, some of the inquiries generated by the language model were similar across different prompts, leading to redundancy and potentially frustrating users by making them sift through repetitive information. To address response repetition, several advanced diversification strategies have been integrated into the system’s framework. Response sampling facilitates the generation of multiple contextually appropriate responses, enabling the selection of the most relevant output based on situational context. Semantic re-ranking further enhances this process by prioritizing responses according to their semantic congruence with the user’s query. Moreover, personalized context modeling leverages historical interaction data and contextual cues to tailor responses, thereby improving relevance and minimizing redundancy. Concerning rate-limit issues, these were identified as technical constraints stemming from external API limitations during model testing. This constraint is extrinsic to the proposed system’s design, reflecting the operational boundaries imposed by third-party service providers. Future implementations could mitigate such limitations through the deployment of self-hosted models or by negotiating enhanced API usage quotas. These enhancements are anticipated to improve system responsiveness, increase output diversity, and support a seamless user experience in real-world applications.

Thus, by introducing a novel prompt evaluation framework, this study contributes significantly to advancing prompt engineering techniques by addressing the critical trade-offs among accuracy, response time, and user satisfaction. The proposed system highlights the potential of optimizing prompt structures to mitigate existing limitations while amplifying system strengths. Notably, exploring a hybrid model that integrates both prompted and non-prompted queries could harness the accuracy benefits of prompted queries while retaining the faster response times of non-prompted approaches. However, the observed decline in SE scores with prompted queries underscores the need to align system responses with user expectations and preferences. Ensuring that the system delivers context-aware, user-centric prompts is imperative for fostering positive user engagement. Additionally, extended response times for prompted queries may pose challenges in real-time applications where immediacy is essential. Therefore, achieving a balanced integration of accuracy and responsiveness remains central to the system’s practical deployment and long-term viability in healthcare settings.

Last but not least, AI-driven healthcare systems will integrate comprehensive measures to address ethical and privacy concerns, ensuring the security of patient data through advanced protocols such as encryption, anonymization, and secure data transmission via SSL and TLS. Encryption will protect data both at rest and during transmission, while anonymization will remove personally identifiable information to maintain patient confidentiality. To address potential algorithmic bias, the AI model will be trained on a diverse dataset encompassing a wide range of demographics, with regular audits conducted to identify and rectify any biases. The system will prioritize transparency and explainability, enabling clinicians to understand the AI’s decision-making processes and ensuring that the technology promotes fair and equitable patient care. Central to the system’s development will be ethical considerations, including informed consent and a patient-centered design approach, with ongoing monitoring and evaluation to adapt to evolving ethical standards and ensure that AI-driven decisions consistently align with the best interests of patients.

## 5 Conclusion

The integration of IoT devices into our AI-driven healthcare system significantly enhances the early detection and management of DED by providing continuous, real-time monitoring of both ocular health and environmental conditions. Key IoT components include smart cameras, environmental sensors, and advanced diagnostic tools, each contributing to a comprehensive assessment of the patient’s condition. Smart cameras capture high-resolution images of the ocular surface, enabling the detection of early signs of DED through advanced image analysis. Environmental sensors monitor factors such as humidity and temperature, correlating these with ocular data to assess the impact of environmental conditions on the progression of DED. Additionally, diagnostic tools like non-invasive tear break-up time analyzers and tear osmolarity meters provide critical quantitative data that inform the AI system’s analysis. This integration allows for dynamic, personalized adjustments to the diagnostic and treatment processes, facilitating timely interventions and improving patient outcomes by preventing the progression of DED.

Amidst the rapid development of generative pre-trained transformers (GPT) and Large Language Models (LLM), their potential is considerable. However, effective utilization hinges on the incorporation of prompts. This study delves into DED, simulating 2,075,747 patient complaints. Employing OpenAI GPT-4.0 and ERNIE Bot-4.0 APIs, a specialized DED-detection-prompt system is established to appraise the urgency of medical attention. The paper underscores the necessity to meticulously balance trade-offs in accuracy, response time, and user satisfaction during the development and deployment of conversational AI systems in medical applications. The study accentuates the pivotal role of prompt engineering in molding user interactions with virtual assistants, particularly within the developmental landscape of ophthalmic GPT. The integration of LLMs, including GPT-based models, in ophthalmic virtual assistant development presents a promising avenue for enriching user interaction in eye healthcare. Future research endeavors should concentrate on optimizing prompt structures, exploring dynamic prompting approaches, prioritizing user-centric evaluations, conducting real-time implementation studies, and contemplating hybrid model development to address identified strengths, weaknesses, opportunities, and threats.

The outlook for Prompt Engineering Strategies in GPT-Based Development appears promising, with ongoing research focusing on precision, context enhancement, and adaptability to contribute to improved user interactions. Emphasizing a user-centric design, future strategies aim to align prompt generation more closely with user preferences and needs. Advancements in fine-tuning techniques and the exploration of hybrid approaches, combining prompt engineering with other methods, offer comprehensive solutions for achieving a balance between accuracy, response time, and user satisfaction. The increasing application of prompt engineering in real-world scenarios, particularly in industries such as healthcare and customer service, underscores its potential impact. Interdisciplinary collaboration between NLP, human-computer interaction, and machine learning continues to drive innovation in prompt engineering. However, addressing ethical considerations, including bias mitigation and user privacy, remains a priority. While challenges persist, the evolving landscape suggests that prompt engineering strategies will play a crucial role in shaping the future of user interaction in GPT-based development.

## Data Availability

The original contributions presented in the study are included in the article/Supplementary material, further inquiries can be directed to the corresponding authors.
