# Supplementary material for: Balancing accuracy and user satisfaction: the role of prompt engineering in AI-driven healthcare solutions
Source: Front Artif Intell. 2025 Feb 13;8:1517918. doi: 10.3389/frai.2025.1517918 (PMC11865202; doi:10.3389/frai.2025.1517918)
Supplement: Supplementary file 1 [file Data_Sheet_1.ZIP › table S1-S5,S6/S6. ethic approval.pdf]

暨南大学珠海临床医学院

珠海市人民医院伦理委员会

科研项目课题申报伦理审查批件

批件号：【2024】-KT-67

项目名称	面向眼科可信算法的神经网络模型可解释性和鲁棒性研究		
项目来源	国家自然科学基金		
本院主要研究者及电话	王涵 19116987045	承接科室	博士后科研工作站
研究类别	☐干预性研究　☑观察性研究<br>☐高风险研究　☐中风险研究　☑低风险研究<br>☐前瞻性研究　☑回顾性研究		
研究目的及意义	神经网络(Neural Network, NN)模型可解释性和鲁棒性一直是医学可信算法及临床应用的关键科学技术难题，二者评价量化难、优化方法不清晰、相互作用关系复杂，平衡策略不明朗。为解决上述难题，本研究选取眼科代表性眼底疾病（老年性黄斑变性）和眼表疾病（干眼症）辅助诊断为应用场景，以图像分类、目标识别和图片分割为主要任务，构建 NN 模型，创新性提出：（1）融合眼科先验知识的神经网络可解释性评价体系和优化策略；（2）神经网络模型鲁棒性评价体系和优化策略；（3）基于可信算法评价体系的神经网络可解释性、鲁棒性相互作用关系研究及平衡策略探讨。神经网络可解释性和鲁棒性技术属于国际先进技术，申请人已积累了充分前期工作。研究为眼科可信算法提供新方法和新视角，为解决神经网络模型黑盒子鲁棒性问题，打破医患使用者不信任、不理解卡脖子问题，助力机器学习在真实世界临床任务中的稳定性和泛化性提升，推动我国数字医疗科学发展和临床应用作贡献，具有显著学术创新和实际应用价值。		
研究起止时间	2024 年 1 月 1 日　至　2027 年 12 月 31 日		
计划入组例数	本研究计划入组 _9000_ 例		
是否涉及体细胞／干细胞	☐是　☑否		
**是否涉及人类遗传资源**<br>*人类遗传资源是指含有人体基因组、基因及其产物的器官、组织、细胞、血液、制备物、重组脱氧核糖核酸（DNA）构建体等遗传材料及相关的信息资料。	研究是否涉及罕见病人群的人类遗传资源采集？ ☐是 ☑否<br>采集的人类遗传资源累积数量是否超过 3000 人？ ☐是 ☑否<br>研究是否为涉及人类遗传资源的国际合作项目？ ☐是 ☑否		

审查决定及意见	☑同意，☐作必要的修正后同意， ☐作必要的修正后重审，☐不同意<br><br>经我院伦理委员会审查，研究者提供的研究信息及内容未违背生命伦理的社会价值，其试验方案符合科学性，试验风险及受试者获益比例合理，研究者能够公平的选择受试者，尊重其权利并使其充分知情，最大程度保护受试者隐私。符合医学伦理的相关规定。<br><br>主任（副主任）委员签名：许劲松<br>珠海市人民医院伦理委员会（盖章）<br>2024年 2 月 28日

Ethics Committee of Zhuhai People's Hospital, Zhuhai Clinical Medical College, Jinan University

Ethical review approval for scientific research projects

Approval number: [2024〗-KT-67

Nape of the neck Eye Name	The interpretability and robustness of the neural network model for ophthalmology trusted algorithms		
Nape of the neck Eye Come	The National Natural Science Foundation of China		
The main researchers and electricity of our institute Words	Wang Han19H6987045	Undertake departmen t	Postdoctoral research workstation
Grind Study intensively Type Don’t	Interventional researchObservational research		
	High-risk research Research on risk in the middle Low-risk research		
	Forward-looking researchRetrospective research		
The purpose and significance of research	Neural networkNeural Network, NNThe interpretability and robustness of the model have always been the key scientific and technological problems of medical credible algorithms and clinical application. The evaluation of the two is difficult to quantify, the optimization method is not clear, the interaction relationship is complex, and the balance strategy is unclear. In order to solve the above problems, this study selects the auxiliary diagnosis of ophthalmic representative fundus diseases (senile macular degeneration) and ocular surface diseases (dry eye) as application scenarios, and takes image classification, target recognition and picture segmentation as the main tasks.NNModel, innovative proposal:(1)Neural network interpretability evaluation system and optimization strategy that integrates ophthalmic prior knowledge; (2) Neural network model robustness evaluation system and optimization strategy; (3Research on the interpretability and robust interaction of neural networks and the discussion of equilibrium strategies based on the trusted algorithm evaluation system. Neural network interpretability and robust technology is an international advanced		

	technology, and the applicant has accumulated sufficient preliminary work. The research provides a new method and a new perspective for the ophthalmic trusted algorithm, in order to solve the problem of the robustness of the neural network model black box, break the problem of distrust and incomprehension of doctors and patient users, help improve the stability and generalization of machine learning in clinical tasks in the real world, and promote the development of digital medical science in China. As a contribution, it has significant academic innovation and practical application value.
The starting and ending time of research	2024Year1Moon1Up to2027Year12Moon31Sun
Planned number of cases	This research plan is to join the group.亠Example
Does it involve somatic cells?/Stem cell	Is it?
Does it involve human genetic resources?<br>*Human genetic resources refer to organs, tissues, cells, blood, preparations, and recombinants that contain human genomes, genes and their products.Deoxyribonucleic acid(DNA)Genetic materials such as constructors and related information materials.	Does the research involve the collection of human genetic resources in rare patients? Yes. Does the cumulative amount of human genetic resources collected exceed?3000People? Yes. Is it an international cooperation project involving human genetic resources? Yes. No.

审查决定及意见	☑同意，☐作必要的修正后同意，☐作必要的修正后重审，☐不同意<br><br>经我院伦理委员会审查，研究者提供的研究信息及内容未违背生命伦理的社会价值，其试验方案符合科学性，试验风险及受试者获益比例合理，研究者能够公平的选择受试者，尊重其权利并使其充分知情，最大程度保护受试者隐私。符合医学伦理的相关规定。<br><br>主任（副主任）委员签名：许[illegible]<br>珠海市人民医院伦理委员会（盖章）<br>2024年 2 月 28日
