# Supplementary material for: Balancing accuracy and user satisfaction: the role of prompt engineering in AI-driven healthcare solutions
Source: Front Artif Intell. 2025 Feb 13;8:1517918. doi: 10.3389/frai.2025.1517918 (PMC11865202; doi:10.3389/frai.2025.1517918)
Supplement: Supplementary file 1 [file Data_Sheet_1.ZIP › table S1-S5,S6/Table S1. English version of OSDI questionnaire.docx]

**Table S1. English version of OSDI questionnaire**

1. Have you had the following symptoms in the past week? Matrix single selection]

|  | It's always like this. | Most of the time/frequent | About half the time | Once in a while | Never |
| --- | --- | --- | --- | --- | --- |
| Eyes are afraid of light. |  |  |  |  |  |
| Sense of foreign object |  |  |  |  |  |
| Eye pain and discomfort |  |  |  |  |  |
| The vision is blurred. |  |  |  |  |  |
| Poor eyesight |  |  |  |  |  |

1. In the past week, have your eyes limited the following aspects of your life? Matrix single selection]

|  | Continue like this. | Most of the time/frequent | About half the time | Once in a while | Never |
| --- | --- | --- | --- | --- | --- |
| Read |  |  |  |  |  |
| Burn the midnight oil |  |  |  |  |  |
| Work with a computer or ATM |  |  |  |  |  |
| Watch TV |  |  |  |  |  |

1. In the past week, when inThe following environmentWill your eyes not live? [ Matrix single selection]

|  | Continue like this. | Most of the time/frequent | About half the time | Once in a while | Never |
| --- | --- | --- | --- | --- | --- |
| When encountering wind and sand |  |  |  |  |  |
| Low humidity area(Very dry) |  |  |  |  |  |
| In an air-conditioned place |  |  |  |  |  |
| When encountering wind and sand |  |  |  |  |  |
