# Supplementary material for: Balancing accuracy and user satisfaction: the role of prompt engineering in AI-driven healthcare solutions
Source: Front Artif Intell. 2025 Feb 13;8:1517918. doi: 10.3389/frai.2025.1517918 (PMC11865202; doi:10.3389/frai.2025.1517918)
Supplement: Supplementary file 1 [file Data_Sheet_1.ZIP › table S1-S5,S6/Table S2. Chinese version of OSDI questionnaire.docx]

**Table S2. Chinese version of OSDI questionnaire**

1. 在过去的一周您是否有过下述症状?[矩阵单选]

|  | 一直这样 | 大部分时间/经常 | 约一半时间 | 偶尔 | 从未 |
| --- | --- | --- | --- | --- | --- |
| 眼睛畏光 |  |  |  |  |  |
| 异物感 |  |  |  |  |  |
| 眼痛不适 |  |  |  |  |  |
| 视物模糊 |  |  |  |  |  |
| 视力不良 |  |  |  |  |  |

1. 在过去的一周，您的眼睛是否限制了你生活的以下方面?[矩阵单选]

|  | 持续这样 | 大部分时间/经常 | 约一半时间 | 偶尔 | 从未 |
| --- | --- | --- | --- | --- | --- |
| 阅读 |  |  |  |  |  |
| 开夜车 |  |  |  |  |  |
| 用电脑或自动取款机工作 |  |  |  |  |  |
| 看电视 |  |  |  |  |  |

1. 在过去的一周，当处于以下环境时您的眼睛会出现不活吗 ?[矩阵单选]

|  | 持续这样 | 大部分时间/经常 | 约一半时间 | 偶尔 | 从未 |
| --- | --- | --- | --- | --- | --- |
| 遇到风沙时 |  |  |  |  |  |
| 低湿度地区(非常干燥)时 |  |  |  |  |  |
| 在有空调的地方 |  |  |  |  |  |
| 遇到风沙时 |  |  |  |  |  |
